# The effects of pyridine hydrochloride on sublethal behavioural endpoints in the common shore crab, *Carcinus maenas*

**DOI:** 10.1007/s10646-025-02993-7

**Published:** 2025-12-04

**Authors:** Elea A. J. Giraud, Alex T Ford

**Affiliations:** Institute of Marine Sciences, University of Portsmouth, Ferry Road, Portsmouth, PO4 9LY United Kingdom

**Keywords:** Keywords: crustaceans, Behaviour assays, Environmental pollution, Shore crabs, Pyridine.

## Abstract

Pyridine is a by-product of several industrial processes and is traditionally extracted from coal tar. In October 2021, a mass stranding of dead, twitching and moribund crustaceans, including edible crabs and lobsters, were observed off the northeast coast of England leading to speculation that pyridine released from dredging was the causal agent. This led to campaign groups asking for a cessation of dredging activity in the Tees estuary until the matter had been resolved. Over the past few years, considerable debate and scepticism have remained among the UK press and the public regarding the potential role of pyridine in this event. To determine the effects of pyridine on juvenile common shore crabs (*Carcinus maenus*) we conducted a four-day static renewal exposure experiment with the nominal concentrations 1, 10, 100 and 1000 µg/L pyridine hydrochloride. Mortality was recorded and novel activity behaviours (Mean Pixel Difference (MSD)) measured using a Zantiks LT machine after 2 h, 24 h, 48 h, 72 h and 96 h. Concentrations were chosen as below those reported to be lethal but represent some environmentally relevant concentrations recorded in the area over the past decade. No mortality was recorded following four-day exposure at concentrations up to 1 mg/L. There was no significant effect of pyridine exposure on the distance travelled by the juvenile crabs during the experiment. Pyridine did not significantly affect the activity of the crabs, as recorded by the MSD analysis. However, there was an interaction between light and exposure where individuals in the highest exposure concentration were significantly more active during light phases of the experiment. No obvious twitching or erratic limb movement was observed over the course of the experiment. This research highlights the minimal effects that pyridine, has on crab movements and activity at environmentally relevant concentrations. This research supports the opinion that pyridine was an unlikely casual factor in the mass mortality event in crustaceans in 2021. Furthermore, we highlight the promising use of MSD for subtle behavioural movement analysis in ecotoxicology.

**Supplementary Information:**

The online version contains supplementary material available at 10.1007/s10646-025-02993-7.

## Introduction

In autumn 2021, large numbers of dead and dying crustaceans washed ashore along 70 km of the northeast coast of England, alarming both members of the public and local fishers (DEFRA 2023a). Dying crabs were reported to be twitching or exhibiting lethargic behaviours, with fishers also reporting an increase in empty crab and lobster pots. The main species which appeared to be affected were the Green shore crab *Carcinus maenas* , Brown crabs *Cancer pagurus* , Velvet swimming crab *Necora puber*, and European lobster *Homarus gammarus* . An interesting observation was that hermit crabs did not appear to be impacted (DEFRA 2023a).

**Fig. 1:**
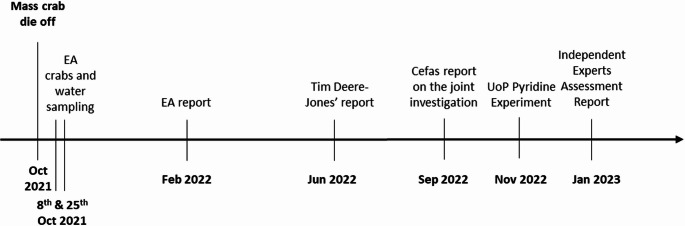
Timeline of events following the UK crustacean mortality event in October 2021

Following this event, the UK Environmental Agency (EA) and the Centre for Environment, Fisheries and Aquaculture Sciences (CEFAS) conducted a series of water, sediment and crab tissue analyses to determine the possible cause of these crustacean deaths (Environmental Agency 2022). The timeline of events is presented in Fig. 1. Although initial theories pointed to an algal bloom, the government agencies were unable to confirm it as a definitive cause (Environmental Agency 2022). The EA detected elevated pyridine in some of the crab tissue samples, but were cautious about drawing conclusions, as their test hadn’t been optimised and validated for crab tissues; additionally, only a few specimens (4 specimens versus 4 controls) had been examined (DEFRA 2023a; Environmental Agency 2022). Despite pyridine concentrations within crabs being on average higher near the location of the mortality event comparisons showed no difference between samples from the mortality events and the reference sites (Ford et al. 2024). Finally, sediment and water analyses in the area, at the time of the die-offs, did not yield any results above detection limits (DEFRA 2023a). The EA has consistently measured pyridine as part of its routine analyses and its presence hasn’t been recorded since 2018 when it was measured at 0.485 µg/L, and the highest measurement recorded at 2.4 µg/L in 2012 (DEFRA 2023a). A subsequent consultant-led review suggested industrial pyridine could be responsible, noting its presence in the TEES sediments and in crab tissues as the only current cause for the mass mortality event along the northeast coast(Deere-Jones 2022).

In October 2022, independent scientists presented further evidence suggesting pyridine as a potential cause of crustacean die-offs, highlighting its high toxicity to decapod crustaceans (Q110 & Q110 of committee transcript; EFRA, 2022). Their pre-print indicated LC50s of 20 mg/L after 24 h and 2.4 mg/L after 72 h in the edible crab *Cancer pagurus* (Eastabrook et al. 2022; preprint), which led the Environment, Fisheries and Rural Affairs committee (EFRA) to request that the Department of Environment, Fisheries and Rural Affairs (DEFRA) to conduct an independent inquiry into the crustacean die-offs. This inquiry later found pyridine was ‘very unlikely’ responsible (DEFRA 2023a), and subsequent CEFAS analyses with optimised methodology showed pyridine levels in affected and unaffected areas were low and not significantly different (DEFRA 2023b). A recent publication by Ford et al. (2024) reviewed the arguments against pyridine being the cause of the mortality event. Despite this, considerable scepticism remains among the UK press and public (Ford et al. 2024).

Teesside has a history of pyridine production, where the chemical has been directly used as a feedstock for the fine-chemical industry and released as a by-product from the iron, steel works and coking industry (DEFRA 2023a). Pyridine (C_5_H_5_N) is a transparent nitrogen-based organic compound, fully soluble in water and highly volatile (Jori et al. 1983). Although first synthesised in 1876 from acetylene and hydrogen cyanide, pyridine was historically produced from coal tar by distillation but is now produced by synthetic processes (Jori et al. 1983; Scriven and Murugan 2000). This organic compound can also be found in natural sources such as the volatile part of black tea or amongst the alkaloid compounds found in the roots of the toxic *Atropa belladonna* plant of the nightshade *Solanaceae* family (Berdai et al. 2012). This compound and its derivatives are often used in the manufacturing of agrochemicals (e.g., bactericides, herbicides), and pharmaceuticals, but can also be used in the production of rubber chemicals or water-repellent agents (Jori et al. 1983). While this compound has also been described to have many biological applications due to its anti-viral, anti-fungicide, anti-cancer and anti-oxidative properties, this compound can also be toxic to humans and animals at high concentrations(Altaf et al. 2015).

Past research shows that exposure to high levels of pyridine derivatives is toxic in mice (Grubb and Oser 1959) or when used via bird baits, can cause lethal consequences to other non-targeted animals such as canine, feline and bovine species (McLean and Khan 2013). Other pyridine-based larvicides used in aquatic environments showed minimal effects on non-targeted aquatic species and low environmental residues (Schaefer and and Miura 1990). However, as derivatives, the effects of these compounds are not directly comparable to pyridine itself. In decapod crustaceans, research is very scarce, with the most recent research being presented by Eastabrook et al. (2022). However, their LC50 modelling has been highlighted for using a low number of replicates (Ford et al. 2024). Therefore, further research is needed to investigate the effect of environmentally relevant concentrations of pyridine on decapods, such as the common shore crab.

During the mass mortality event, members of the public reported observing a distinct twitching behaviour in dying crabs along the shoreline. Twitching was also reported with pyridine exposure according to Eastabrook et al. (2022). Comparable behaviours, though described as “shaking,” have been documented in *Carcinus maenas* crabs following exposure to acetic acid (Barr and Elwood 2024). Similarly, involuntary movements including shaking and twitching, have been observed across multiple crab species exposed to various toxic substances. For instance, Rainwater (Rainwater 2002) described these behaviours in moribund ghost crabs (*Ocypode quadrata* Fabricius) exposed to carbamate (CA) pesticides, while Dyuizen et al.(2012) observed similar reactions in *Hemigrapsus sanguineus* crabs when exposed to formalin. Similarly, bacterial, viral and protozoan pathogens are known to induce twitching which are sometimes referred to as ‘tremor disease’ in some decapod crustaceans (reviewed in (DEFRA 2023a). These findings suggest that, while similar twitching behaviours have been observed in the literature, further research is needed to determine whether pyridine could cause a twitching response in crabs at environmentally relevant concentrations.

Behavioural toxicology has expanded rapidly over the past few decades (Bertram et al. 2025). While some tracking software offer the ability to track an object by focussing on a fixed point Zantiks software (zantiks.com) offers the ability to measure movement by looking for changes in Mean Pixel Difference (MSD). This method offers advantages where an animal might be stationary but still moving their appendages (i.e. twitching), by detecting pixel changes between successive image frames. It provides an opportunity to study subtle movement behaviours such as twitching when the crabs are actively moving limbs but not tracked around the experimental arena, which are not detectable through positional tracking alone. This study used the MSD approach, along with movement tracking, to identify changes in behaviour and subtle movements.

This study does not aim to establish the underlying causes of the crab mass mortality event in October 2021 but rather to determine the lethal and sublethal effects of pyridine on juvenile common shore crabs (*Carcinus maenus*). Juvenile crab individuals were chosen in this study because they can be reliably and simultaneously tracked at this stage using the Zantiks software. Furthermore, prior research on mass mortality events suggests that juvenile crabs may be particularly sensitive to chemical exposure in estuaries (Osterberg et al. 2012).This was achieved by determining the effects of short-term pyridine exposure on the survivorship and behaviour of common shore crab *Carcinus maenus* following 4 days of exposure at environmentally relevant concentrations. It was hypothesised that acute pyridine exposure would (i) increase mortality, (ii) induce abnormal behaviour such as twitching and, (iii) affect activity levels measured by distance travelled and mean square difference analysis (MSD).

## Methods

### Animal collection and husbandry

60 juvenile common shore crabs *Carcinus maenas* were sampled off the coast at Langstone Harbour (50°49′04″N, 001°00′52″W) in Portsmouth, UK. Crabs ranged between 7.38 mm and 22.40 mm and were randomly assigned between concentration groups. 10 crabs were euthanised for analysis of environmental levels prior the start of the experiment. 50 crabs (13.6 ± 3.4 mm) were placed into microcosms filled with aerated artificial seawater (10 °C, 32 ppt). Microcosms were placed into a 10 °C incubators Individuals were acclimated for 7 days. Escapes (*n* = 5 overall) were excluded from the experimental analyses.

### Exposure and tracking protocol

After the acclimation phase, individuals were distributed to one of the five nominal concentrations of pyridine: control, 1, 10, 100 and, 1000 µg/L. Stock solution of pyridine dissolved in artificial seawater were prepared on the day of exposure using pyridine hydrochloride (SIGMA ALDRICH -CAS number 628-13-7). *C. maenas* crabs were exposed once for a total of 96 h during acute toxicity assays and during which moults and deaths were recorded, and after which crabs were euthanised.

**Fig. 2:**
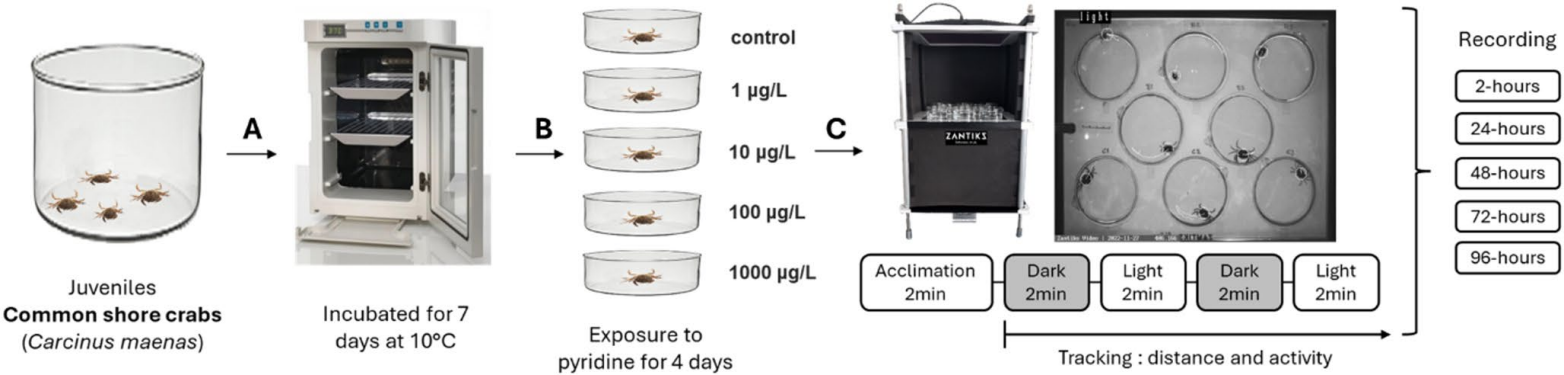
Schematic of experiment design showing the acclimation phase **A**, exposure to chosen concentrations **B** and the protocol of tracking system method **C**

During the exposure, distance moved (mm) and activity were measured using a Zantiks LT machine after 2 h, 24 h, 48 h, 72 h and 96 h (Fig. 2). Activity was measured using pixel change and Mean Square Difference (MSD). Videos were also visually inspected to identify the occurrence of twitching.

### Data analysis

Statistical analyses were performed using R (R Core Team 2025)(R Core Team 2025)(R Core Team 2025)(R Core Team 2025). Distance travelled and activity were calculated for each 2-minute time bin. Extreme outliers were calculated (values > Q3-3*IQR) and removed from the data analysis. The count of moults observed over exposure time and treatments were analysed using a Generalised Linear Mixed-effects Model (GLMM) with the Poisson family. Comparison of distance travelled and activity between treatments were performed using Linear Mixed-effects Models (LMM). Treatment, time and crab size (mm) were set as fixed factors, while crab ID was set as a random factor to account for individual variability and repeated measures. Anomalous values occurred when tracking was lost by the Zantiks software. The most appropriate model was selected using the AIC criterion. Models fit and normality were checked using QQ and fitted versus residual plots. Generalised linear mixed model assumptions were assessed for overdispersion;; diagnostics do not indicate over- or under dispersion. Results are reported with effect sizes and with 95% confidence Intervals (CI).

## Results

No mortality was recorded throughout the 4-day experiment in any of the control or exposed groups. A few moults were observed in some exposure groups but no significant differences in the overall moulting frequency were observed over time (Poisson GLMM, IRR = 1.29, 95% CI = 0.63–2.66; *p* = 0.486) or between control and exposed groups (Poisson GLMM, IRR = 1.00, 95% CI = 0.99–1.01, *p* = 0.501; Table 1).

**Table 1:** Number of replicates used for each concentration of exposure, recorded mortality and moults

Concentration	Replicates	Mortality (%)	Moults (%)
Control	9	0	0
1 µg/L	8	0	12.5
10 µg/L	10	0	20
100 µg/L	10	0	10
1000 µg/L	8	0	0

### Distance travelled

**Fig. 3:**
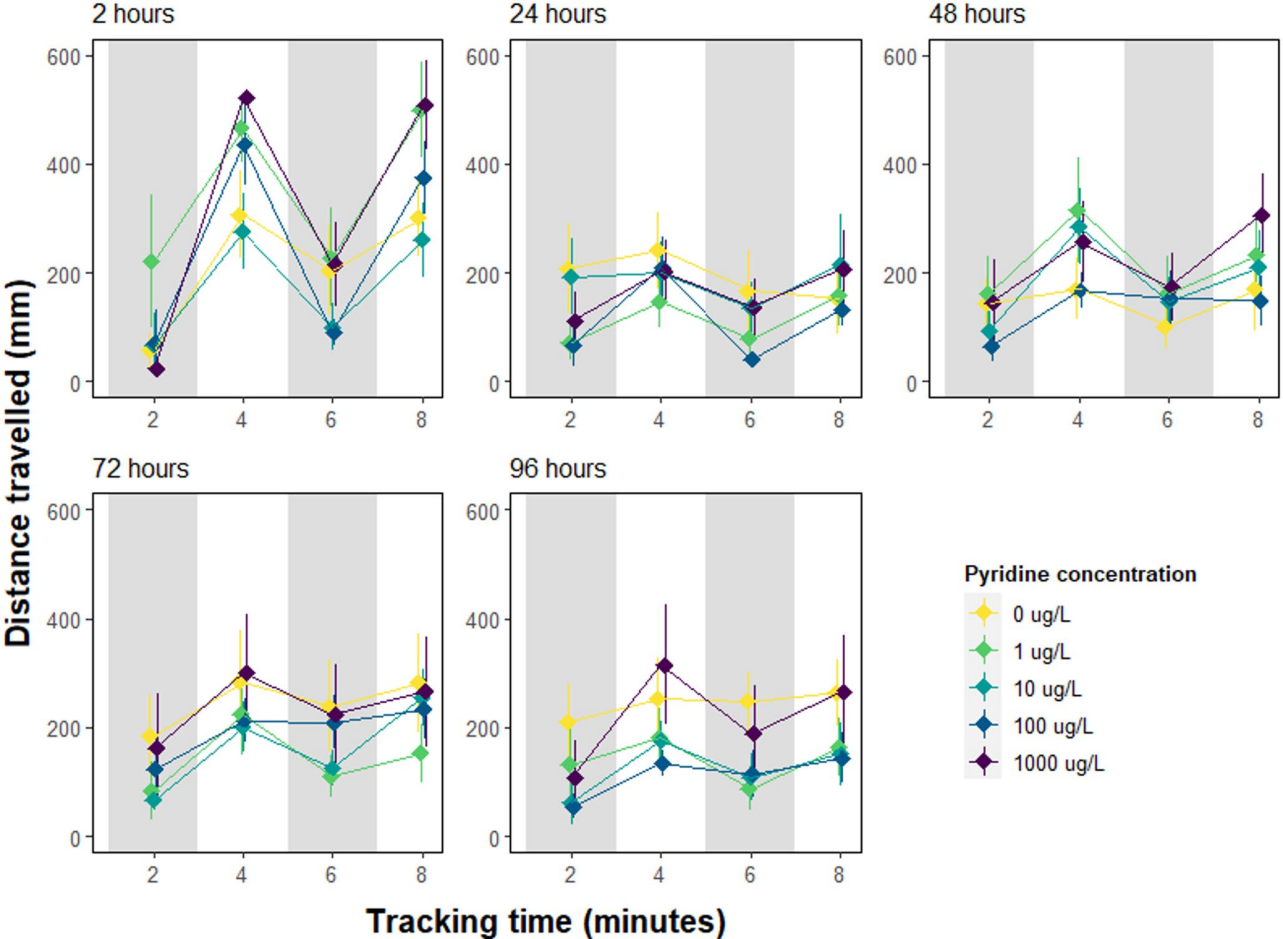
Distance travelled (mm ± se) by juvenile *Carcinus maenas* crabs (*n* = 45) exposed to 0, 1, 10, 100 and 1000 µg/L of Pyridine Hydrochloride. Distance travelled in crystalline dishes was recorded after 2, 24, 48, 72 and 96 h of exposure for a total tracking duration of 8 min

Pyridine exhibited no effect on the distance travelled by crabs during the exposure trials (LMM, β = 0.02, 95% CI= -0.08–0.12, *p* = 0.721, Appendix Table 1). There was a significant increase in the distance travelled by juvenile crabs in the light stimuli (LMM, β = 168.48, 95% CI = 122.41–214.56, *P* < 0.001, Appendix Table 1), this response changed significantly with the experimental duration time, such that as the time (days) increased, the effect of the light condition decreased (Fig. 3; LMM, β=-31.28, 95% CI=-50.08 – -12.47, *P* < 0.001). The individual’s size was a significant factor in the distance travelled, such that larger individuals covered smaller distances (LMM, β=-15.30, 95% CI=-23.38 – -7.21, *p* < 0.001). The variance for random intercept and residual variance were large suggesting the substantial variance between individual crabs and unexplained variability in the model.

### Activity (MSD)

**Fig. 4:**
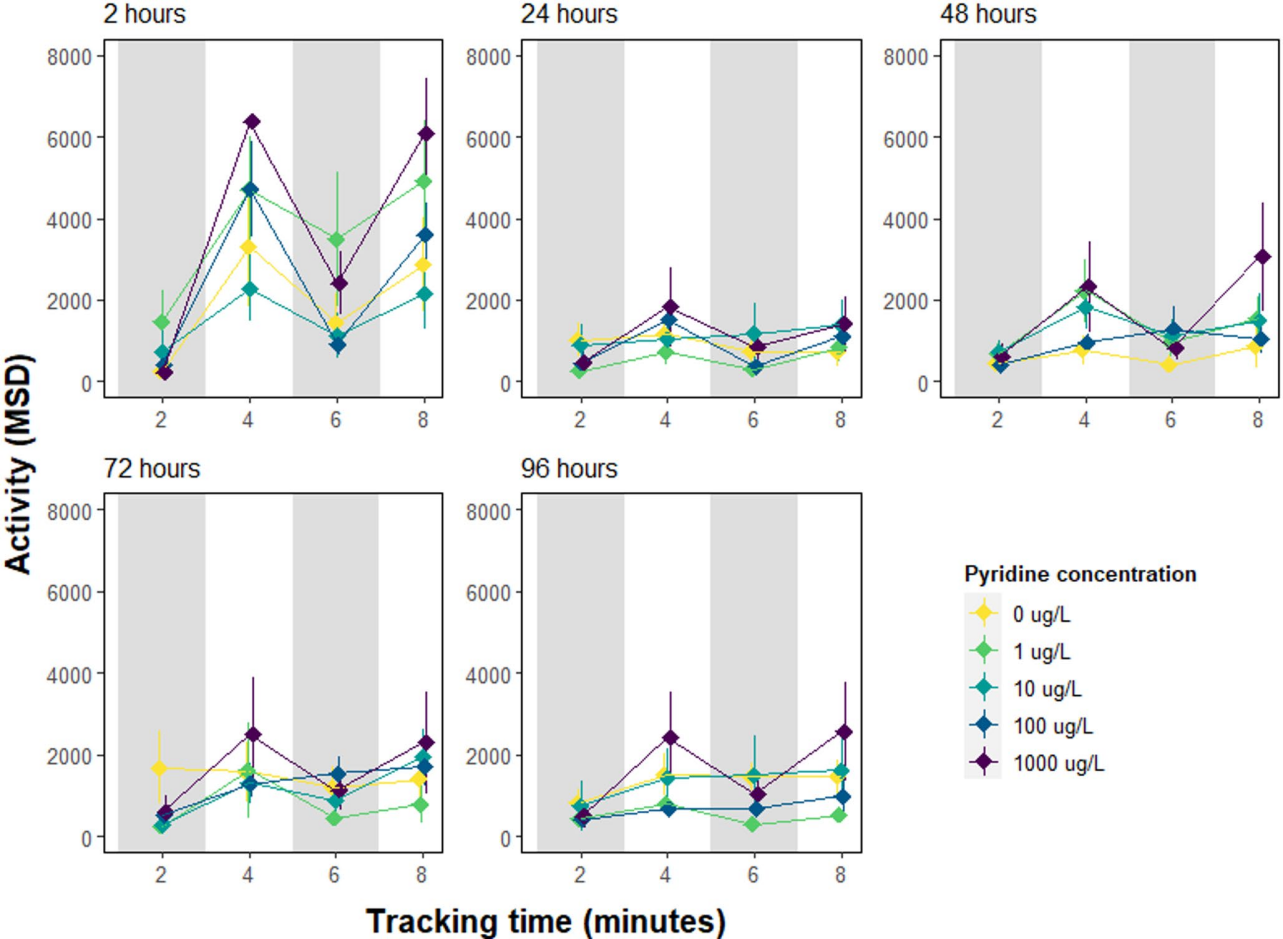
Mean activity (± se) of juvenile *Carcinus maenas* crabs (*n* = 45) exposed to 0, 1, 10, 100 and 1000 µg/L of Pyridine Hydrochloride. *C. maenas* were exposed to Pyridine hydrochloride in crystalline dishes for 96 h and activity (Mean Square Distance) was recorded after 2, 24, 48, 72 and 96 h for a total tracking duration of 8 min. Shaded areas display the dark phases

The pixel mean square difference (MSD) was used to determine if the exposure to pyridine hydrochloride increased the activity of juvenile *Carcinus maenas* crabs exposed to alternating phases of dark and light (Fig. 4). This allowed the assessment of behaviours, such as limb movement (twitching), which may not result in increased or decreased distance covered. There was no overall effect of concentration on the activity of the crabs (LMM, β = 0.20, 95%CI = -0.82–1.21, *P* = 0.705, Appendix Table 2). However, there was an interaction between concentration and the light-dark phases of the experiment (LMM, β = 1.71, 95%CI = 0.53–2.88, *P* < 0.005). The activity of crabs was increased during phases of light stimuli (LMM, β = 1467.22, 95%CI = 967.89–1966.55, *P* < 0.001, Appendix Table 2), although the effect of the light stimulus decreased over the exposure period (LMM, β=-333.51, 95%CI= -537.36 – -129.66, *P* < 0.001). The activity of individuals during light stimuli phases was increased for individuals exposed to all pyridine concentrations (LMM, *P* < 0.005, Appendix Table 2) and for larger individuals (LMM, β = 124.73, 95%CI = 50.58–198.87, *P* < 0.001).

**Fig. 5:**
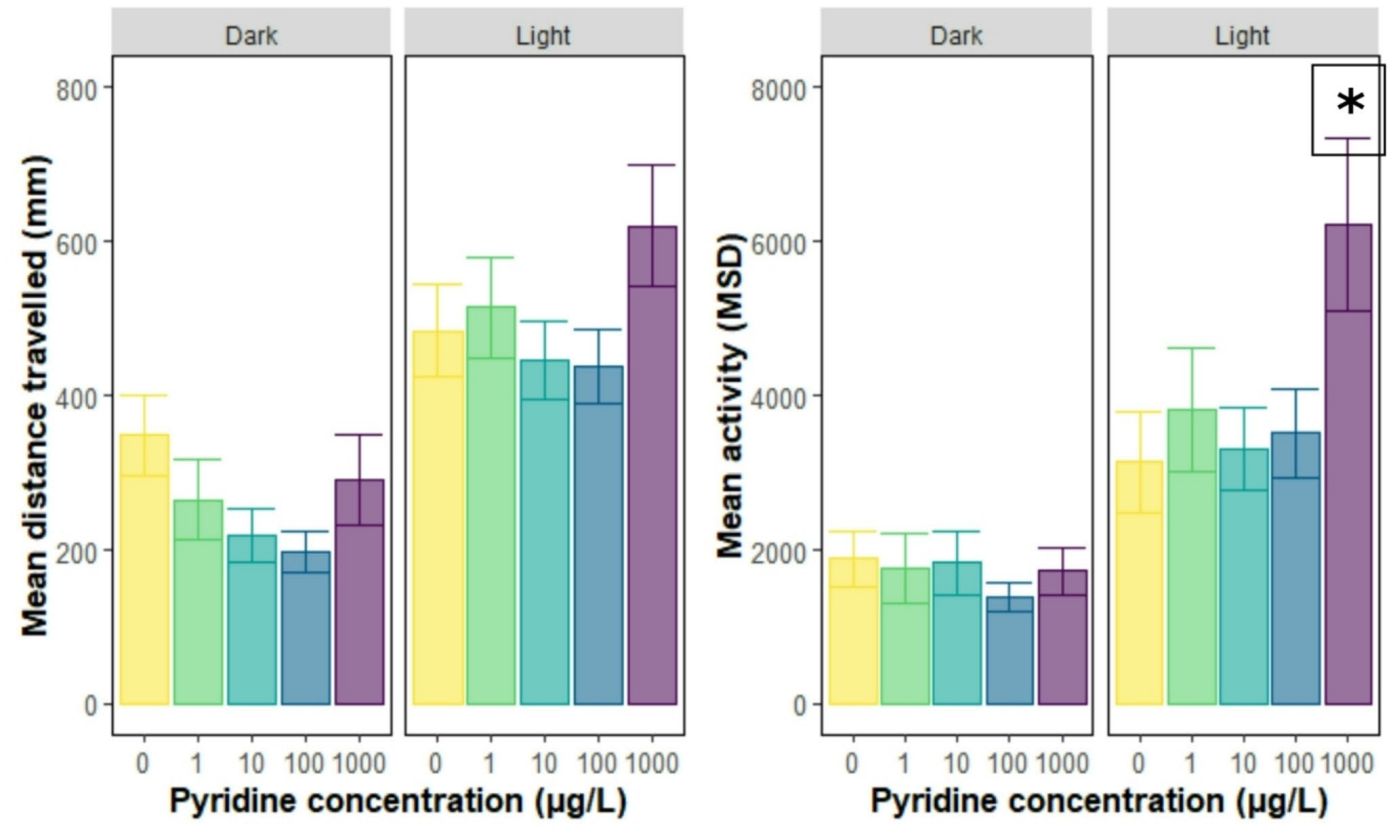
Mean (± se) distance travelled and activity per light stimuli during a trial of juvenile *Carcinus maenas* crabs exposed to 0, 1, 10, 100 and 1000 µg/L of pyridine hydrochloride. *C. maenas* were exposed to Pyridine hydrochloride in crystalline dishes for 96 h and activity (Mean Square Distance) was recorded after 2, 24, 48, 72 and 96 h for a total tracking duration of 8 min. Shaded areas display the dark phases

The interaction between the light stimuli and concentration showed that the effect of concentration varies whether crabs are exposed to light therefore suggesting a modulating effect of light on how concentration impacts activity (LMM, *p* < 0.001), where individuals exposed to the highest concentrations of Pyridine were more likely to show signs of increased activities in presence of the light (Fig. 5, Appendix Table 3).

Finally, crab sizes (mm) also affected the total distance travelled and activity of juveniles but in opposite directions. The size of the crabs negatively influenced their total distance travelled, with larger crabs travelling shorter distances in total (LMM, β=-30.53, 95%CI= -46.38 – -14.68, *p* < 0.001, Appendix Table 4). Size had a positive effect on total activity, where larger crabs exhibited higher activity levels than smaller ones (LMM, β = 249.45, CI = 100.96–397.95, *P* = 0.001, Appendix Table 5).

Visual inspection of the recorded crab movement recording did not reveal any signs of twitching behaviour. Instead, the crabs exhibited normal movement patterns. Their observed behaviours included using their claws to interact with their mouths and employing various legs movement while moving around, but their limbs remained relatively still while stationary, with occasional limb movement. Notably, there was no indication of rapid limb movement or twitching behaviour throughout the recordings.

## Discussion

Pyridine has been proposed as the causal factor in a large-scale crustacean mortality event in the northeast of England, resulting in the mass death of tens of thousands of crabs and lobsters (Deere-Jones 2022; Eastabrook et al. 2022). In response to claims that pyridine was extremely toxic to crustaceans and induced twitching behaviours, the effect of Pyridine on common shore crab mortality and activity behaviours were studied.

The highest ever recorded concentration of pyridine in the Tees Estuary was 2.4 µg/L in 2012 when it was discharged as effluent under license from the EA. Our study failed to record a single mortality in crabs exposed to environmentally relevant concentrations between 1 µg/L and 1000 µg/L over a 4-day exposure period. Eastabook et al. (2022) recorded LC50s of 19.44 mg/L after 24 h and 2.75 mg/L after 72 h. The highest concentration in our study (1 mg/L) was lower than the lowest concentration (2 mg/L) used by Eastabrook et al. (2022). Therefore, differences in the studies could reflect differences in sensitivities between edible crab (*Cancer pagarus*) and common shore crabs *(C. maenas)* or the concentration ranges used. However, both these species were impacted during the mortality event. The calculated LC50s in Eastabrook et al. (2022) were below their calculated No Observed Effect Concentrations (NOECs) and may have been inaccurately estimated due to small sample sizes (Ford et al. 2024). LC50s in crustaceans from the published literature range from 50 to 2550 mg/L (US EPA Knowledgebase; Olker et al. 2022, Slooff 1983) which suggests our experimental concentrations, despite being more environmentally relevant, were markedly below all other mortality studies with crustaceans. However, given that pyridine concentrations have never been reported close to lethal concentrations (> 50 mg/L) prior to or after the mortality event (DEFRA 2023a), this current research supports the evidence that pyridine is highly unlikely as a causal factor in the mass mortality event of October 2021.

The results of our behavioural experiments indicate a strong and consistent effect of light exposure on the distance travelled and activity of crabs over time, with crabs being more active and covering greater distances during light phases of the experiment. However, pyridine concentrations had no significant effect on the distance travelled by the crabs and no interaction between concentrations with light or exposure length. To date, there are very few studies on the behavioural impacts of pyridine exposure, and none recorded on the common shore crab. Light or visual cues are known to affect feeding, hiding or movement behaviour in adults and larval stages of crustaceans (Fort and Tulloch 2016; Ping et al. 2015; Shirley and Shirley 1988). However, the factors contributing to the increased response to light stimuli in animals exposed to higher concentrations remain unknown and require further investigation.

We did not find evidence of ‘twitching’, erratic limb movements, nor any other similar behaviour such as ‘shaking’ as described by members of the public during the mass die-off and in previous crustacean research (Dyuizen et al. 2012; Eastabrook et al. 2022; Rainwater 2002). Despite not finding a significant effect of pyridine exposure on crab activity directly, we did observe a significant interaction between concentration and light phases using the MSD analysis. This was driven by crabs being more active in the highest concentration during the light and not dark phases of the experiment. Given there was no significant difference in distance covered but there was an increase in activity, this might indicate increased leg movements recorded by the Mean Pixel Different (MSD) analysis. Further research would therefore needed to determine whether longer-term exposure or higher concentrations would induce visible twitching symptoms.

While we did not observe any significant effect of pyridine on the behaviour of common shore crabs, some limitations are discussed. One notable limitation in this study was the use of nominal concentrations which likely diminished throughout the 4-day experimental period. The environmental residence time of pyridine is considered to be short; recorded to last only days in the water column and months in sediments (Sims et al. 1989). Additionally, crab size was found to be a significant confounding variable influencing both distances travelled and overall crab activity, suggesting that more careful selection of test species may help minimise intra-species variability of behavioural endpoints. Finally, individuals across all treatment groups demonstrated a decrease in activity over time (see Figs. 3 and 4) suggesting that prolonged exposure may gradually reduce activity through habituation to experimental conditions. Given its highly volatile nature (Sims et al. 1989), pyridine is likely to dissipate rapidly from the environment.

This study contributes valuable insights to the limited knowledge on the effect of pyridine on aquatic organisms and specifically decapod crustaceans such as *Carcinus maenas*. This research showed that no mortality was recorded during the 4-day exposure period to pyridine (up to 1000 µg/L) and no effects of pyridine were observed on distance travelled, although an interaction between light phase and pyridine concentrations was measured for crab activity at the highest concentration. No obvious signs of twitching behaviour were recorded by human observation, but the use of Mean Pixel Different (MSD) analysis presents a promising method in addition to tracking single points when measuring subtle animal behaviours in ecotoxicology.

## Supplementary Information

Below is the link to the electronic supplementary material.

Supplementary Material 1

## Data Availability

Data generated on this manuscript is available via a University of Portsmouth repository.
