# Supplementary material for: The effects of pyridine hydrochloride on sublethal behavioural endpoints in the common shore crab, Carcinus maenas
Source: Ecotoxicology. 2025 Dec 4;35(1):4. doi: 10.1007/s10646-025-02993-7 (PMC12678539; doi:10.1007/s10646-025-02993-7)
Supplement: Supplementary file 1 — Supplementary Material 1 [file 10646_2025_2993_MOESM1_ESM.docx]

**The effects of pyridine hydrochloride on behaviour in the common shore crab, *Carcinus maenas***

Elea AJ Giraud^1^, Alex T Ford^1#^

^1^Institute of Marine Sciences, University of Portsmouth, Ferry Road, Portsmouth, PO4 9LY

^#^Corresponding author: alex.ford@port.ac.uk

**Appendices**

**Appendix Table 1:** **Summary output of the Linear Mixed-Effect Model assessing the distance travelled by juvenile Carcinus maenas crabs (n=45) exposed to pyridine hydrochloride under alternating light and dark conditions.** Distance travelled was measured in two-minute time-bin intervals. C. maenas crabs were exposed to pyridine hydrochloride for four days at concentrations of 0 μg/L (control), 1, 10, 100, and 1000 μg/L.

|  | **Total distance per 2-min time-bins** | | |
| --- | --- | --- | --- |
| ***Predictors*** | ***Estimates*** | ***CI*** | ***p*** |
| **(Intercept)** | 338.08 | 219.84 – 456.32 | **<0.001** |
| **Light Condition [Light]** | 168.48 | 122.41 – 214.56 | **<0.001** |
| **Concentration** | 0.02 | -0.08 – 0.12 | 0.721 |
| **Day** | 3.33 | -9.99 – 16.65 | 0.624 |
| **Crab size (mm)** | -15.30 | -23.38 – -7.21 | **<0.001** |
| **Light Condition [Light] × Concentration** | 0.08 | -0.03 – 0.19 | 0.167 |
| **Light Condition [Light] × Day** | -31.28 | -50.08 – -12.47 | **0.001** |
| **Concentration × Day** | 0.01 | -0.02 – 0.04 | 0.630 |
| **(Light Condition [Light] × Concentration) × Day** | -0.01 | -0.05 – 0.04 | 0.776 |
| **Random Effects** | | | |
| **σ^2^** | 31783.39 | | |
| **τ_00_ _crab.id_** | 7261.55 | | |
| **ICC** | 0.19 | | |
| **N _crab.id_** | 45 | | |
| **Observations** | 897 | | |
| **Marginal R^2^ / Conditional R^2^** | 0.167 / 0.322 | | |

**Appendix Table 2: Summary output of the Linear Mixed-Effect Model evaluating the activity of juvenile Carcinus maenas (n=45) crabs exposed to pyridine hydrochloride under alternating light and dark conditions.** Activity, measured as mean pixel change (MDS), was recorded in two-minute time bins. C. maenas crabs were exposed for four days to pyridine hydrochloride at concentrations of 0μg/L (control), 1, 10, 100, and 1000 μg/L.

|  | **Total activity per 2-min time-bins** | | |
| --- | --- | --- | --- |
| ***Predictors*** | ***Estimates*** | ***CI*** | ***p*** |
| **(Intercept)** | -720.53 | -1818.02 – 376.96 | 0.198 |
| **Light Condition [Light]** | 1467.22 | 967.89 – 1966.55 | **<0.001** |
| **Concentration** | 0.20 | -0.82 – 1.21 | 0.705 |
| **Day** | -44.07 | -188.21 – 100.08 | 0.549 |
| **Crab size (mm)** | 124.73 | 50.58 – 198.87 | **0.001** |
| **Light Condition [Light] × Concentration** | 1.71 | 0.53 – 2.88 | **0.005** |
| **Light Condition [Light] × Day** | -333.51 | -537.36 – -129.66 | **0.001** |
| **Concentration × Day** | -0.05 | -0.39 – 0.29 | 0.761 |
| **(Light Condition [Light] × Concentration) × Day** | -0.10 | -0.58 – 0.38 | 0.693 |
| **Random Effects** | | | |
| **σ^2^** | 3749892.55 | | |
| **τ_00_ _crab.id_** | 557008.06 | | |
| **ICC** | 0.13 | | |
| **N _crab.id_** | 45 | | |
| **Observations** | 900 | | |
| **Marginal R^2^ / Conditional R^2^** | 0.163 / 0.271 | | |

**Appendix Table 3*:* Pairwise comparison of the total activity between concentrations of Pyridine (µg/L) within each Light Condition, based on Estimated Marginal Means (EMMs) from a Linear Mixed-Effects.** The model included fixed effects for light condition, concentration, exposure time (referred to as days), and crab size, with crab ID as random effects. Comparisons are expressed as differences between of total activity, with standard errors, degrees of freedom, t-ratios and adjusted p-values.

| **Pairwise Comparison** | **Light Condition** | **Estimate** | **SE** | **DF** | **t-value** | **p-value** |
| --- | --- | --- | --- | --- | --- | --- |
| **0 µg/L (Control) vs 1 µg/L** | Dark | 554.5248 | 1032.1652 | 78.1523 | 0.5372 | 0.9832 |
| **0 µg/L (Control) vs 10 µg/L** | Dark | 642.6076 | 979.9979 | 77.6407 | 0.6557 | 0.9651 |
| **0 µg/L (Control) vs 100 µg/L** | Dark | 958.6816 | 976.1895 | 78.1269 | 0.9821 | 0.8626 |
| **0 µg/L (Control) vs 1000 µg/L** | Dark | 308.5001 | 1031.3081 | 78.2572 | 0.2991 | 0.9982 |
| **1 µg/L vs 10 µg/L** | Dark | 88.0828 | 1005.5915 | 78.4031 | 0.0876 | 1.0000 |
| **1 µg/L vs 100 µg/L** | Dark | 404.1568 | 1004.8701 | 78.4945 | 0.4022 | 0.9944 |
| **1 µg/L vs 1000 µg/L** | Dark | -246.0247 | 1059.3016 | 78.4854 | -0.2323 | 0.9993 |
| **10 µg/L vs 100 µg/L** | Dark | 316.0740 | 948.2268 | 78.3835 | 0.3333 | 0.9973 |
| **10 µg/L vs 1000 µg/L** | Dark | -334.1074 | 1006.1551 | 78.3320 | -0.3321 | 0.9973 |
| **100 µg/L vs 1000 µg/L** | Dark | -650.1815 | 1004.9320 | 78.4866 | -0.6470 | 0.9667 |
| **0 µg/L (Control) vs 1 µg/L** | Light | -69.5319 | 1032.1652 | 78.1523 | -0.0674 | 1.0000 |
| **0 µg/L (Control) vs 10 µg/L** | Light | 457.1330 | 979.9979 | 77.6407 | 0.4665 | 0.9901 |
| **0 µg/L (Control) vs 100 µg/L** | Light | 107.2072 | 976.1895 | 78.1269 | 0.1098 | 1.0000 |
| **0 µg/L (Control) vs 1000 µg/L** | Light | -3011.6693 | 1031.3081 | 78.2572 | -2.9202 | **0.0359** |
| **1 µg/L vs 10 µg/L** | Light | 526.6649 | 1005.5915 | 78.4031 | 0.5237 | 0.9847 |
| **1 µg/L vs 100 µg/L** | Light | 176.7391 | 1004.8701 | 78.4945 | 0.1759 | 0.9998 |
| **1 µg/L vs 1000 µg/L** | Light | -2942.1374 | 1059.3016 | 78.4854 | -2.7774 | 0.0519 |
| **10 µg/L vs 100 µg/L** | Light | -349.9258 | 948.2268 | 78.3835 | -0.3690 | 0.9960 |
| **10 µg/L vs 1000 µg/L** | Light | -3468.8023 | 1006.1551 | 78.3320 | -3.4476 | **0.0079** |
| **100 µg/L vs 1000 µg/L** | Light | -3118.8765 | 1004.9320 | 78.4866 | -3.1036 | **0.0217** |

**Appendix Table 4: Summary output of the Linear Mixed-Effect Model assessing the total distance travelled by juvenile Carcinus maenas crabs (n=45) exposed to pyridine hydrochloride under alternating light and dark conditions.** Distance travelled was measured over the total tracking trial. C. maenas crabs were exposed to pyridine hydrochloride for four days at concentrations of 0 μg/L (control), 1, 10, 100, and 1000 μg/L.

|  | **Total distance per trial** | | |
| --- | --- | --- | --- |
| *Predictors* | *Estimates* | *CI* | *p* |
| (Intercept) | 687.05 | 460.16 – 913.94 | **<0.001** |
| L CONDITION [Light] | 213.45 | 144.99 – 281.91 | **<0.001** |
| concentration | 0.06 | -0.11 – 0.23 | 0.477 |
| size mm | -30.53 | -46.38 – -14.68 | **<0.001** |
| L CONDITION [Light] × | 0.14 | -0.02 – 0.30 | 0.097 |
| concentration |  |  |  |
| **Random Effects** | | | |
| σ^2^ | 105451.09 | | |
| τ_00_ _crab.id_ | 23391.71 | | |
| ICC | 0.18 | | |
| N _crab.id_ | 45 | | |
| Observations | 450 | | |
| Marginal R^2^ / Conditional R^2^ | 0.180 / 0.329 | | |

**Appendix Table 5: Summary output of the Linear Mixed-Effect Model evaluating the total activity of juvenile Carcinus maenas (n=45) crabs exposed to pyridine hydrochloride under alternating light and dark conditions.** Activity, measured as mean pixel change (MDS), was recorded over the total duration of the tracking trial. C. maenas crabs were exposed for four days to pyridine hydrochloride at concentrations of 0μg/L (control), 1, 10, 100, and 1000 μg/L.

| **Total activity per trial** | | |  |
| --- | --- | --- | --- |
| *Predictors* | *Estimates* | *CI* | *p* |
| (Intercept) | -1617.33 | -3753.64 – 518.99 | 0.137 |
| L CONDITION [Light] | 1600.39 | 827.41 – 2373.36 | **<0.001** |
| concentration | 0.18 | -1.45 – 1.82 | 0.826 |
| size mm | 249.45 | 100.96 – 397.95 | **0.001** |
| L CONDITION [Light] × | 3.03 | 1.21 – 4.85 | **0.001** |
| concentration |  |  |  |
| **Random Effects** | | | |
| σ^2^ | 13442455.87 | | |
| τ_00_ _crab.id_ | 1633764.99 | | |
| ICC | 0.11 | | |
| N _crab.id_ | 45 | | |
| Observations | 450 | | |
| Marginal R^2^ / Conditional R^2^ | 0.150 / 0.242 | | |
